# Evaluating the effectiveness of public hospital reform from the perspective of efficiency and quality in Guangxi, China

**DOI:** 10.1186/s40064-016-3598-y

**Published:** 2016-11-04

**Authors:** Shuai Jiang, Wei-min Wu, Pengqian Fang

**Affiliations:** 1 School of Medicine and Health Management, Tongji Medical College, Huazhong University of Science and Technology, 13 Hangkong Road, Qiaokou District, Wuhan, 430030 Hubei China; 2 Academy of Health Policy and Management, Huazhong University of Science and Technology, 13 Hangkong Road, Qiaokou District, Wuhan, 430030 Hubei China; 3 School of Information and Management, Guangxi Medical University, 22 Shuangyong Road, Qingxiu District, Nanning, 530021 Guangxi China; 4 Guangxi Institute for Food and Drug Control, 9 Qinghu Road, Qingxiu District, Nanning, 530021 Guangxi China

**Keywords:** Public hospital reform, Effectiveness evaluation, Patient satisfaction, Clinical terminal quality, Data envelopment analysis

## Abstract

The evaluation of hospital reform effectiveness in health care reform is very important and beneficial since it helps the government to understand the current situation of pilot county public hospitals and smoothly start the reform in all county hospitals. This study used sample hospitals data from 2010 to 2012 to evaluate the effectiveness of public hospital reform through comparisons between 2010 and 2012 in hospital operating efficiency, clinical terminal quality, the average medical expense of patients, patient and medical staff overall satisfaction. The results highlight that there was no improvement in hospitals’ operating efficiency, pilot hospitals’ clinical terminal quality was lower than its counterpart, and the two objectives had been improved: medical expense control and inpatients’ satisfaction, however, nearly 68.46% medical staffs were dissatisfied with the outcomes of the reform in pilot hospitals staff satisfaction. Nevertheless, the reform was helpful to improve the hospital current situation with some of the objectives having been achieved. It has not completely succeeded in Guangxi, China.

## Background

In March 2009, the Chinese government has launched formally Healthcare Reform with a focus on gradually achieving the goal, for example, everyone has access to basic medical care and health services, as well as improving medical and health services at the grassroots level. The reform of county public hospitals is one of the major tasks in medical reform, and the next plan is to establish a modern hospital management system, eliminating drug price competition and standardizing referral procedures. The government rolled out an 850 billion yuan (130 billion U.S. dollar) 3-year plan for health care reform, and 70 billion yuan of the plan was invested to county public hospitals.

Guangxi Zhuang Autonomous Region of China (Guangxi), a minority area in southern China, is an ethnically diverse region containing 12 major ethnic groups such as Zhuang, Yao, Miao, Dong, Mulao, Maonan, Hui, Bouyei, Jing, Shui, Yi minority and Han majority group and so on. The majority of whom reside in the mountainous regions (Zhang et al. 2016), where traffic are very inconvenient, and to some extent, this type of terrain has restricted local social and economic development. That is to say, the service ability and level of local medical institution didn’t meet people’s growing health needs. It was very crucial to promote the sustainable development of medical institutions by increase health investment for improving the accessibility, utilization and quality of services.

Governments at all levels increased health investment of each county to improve infrastructure, health workforce, medical equipment, discipline construction, salary and reward of medical workers, basic pharmaceuticals policy, basic medical insurance and so on. In 2011, a financial support with 14 million yuan was launched to start the first pilot county public hospital reform in seven counties: Wuming, Luzhai, Xing’an, Shangsi, Rong, Tiandong and Yongfu, which are located in the east, south, west, north and center of Guangxi. During 2010–2012, those seven counties’ hospitals developed significantly: the number of beds and medical staffs in county hospitals increased by 5.46 and 8.86%, reaching 3122 and 4123, and the number of outpatients and inpatients in county hospitals increased by 12.27 and 14.43%, reaching 2356.86 and 160.55 thousand.

In China, some researchers evaluated the reform effectiveness from different perspectives. Some studies showed that operational efficiency and productivity of county hospital have gone down (Ying 2015; Jiang et al. 2015; Shuai 2015), and patient’s medical expenses and cost of medicine have fallen to some extent as health reform starts (Huimei 2014; Shuai et al. 2014; Zhenfang 2014). Some studies also showed that patients’ satisfaction were not throughout improved with the implementation of the reform (Suyi et al. 2013; Weiyun and Yulan 2014), some medical staffs who exhibited a better understanding of the public hospital reform program hold negative comments about the reform effectiveness in pilot county hospitals (Fang et al. 2015; Dongmei et al. 2015). Additionally, the scale of the overall liabilities of the county general hospitals was expanding with large differences among regions, with well-developed short-term debt repaying capacity, but the long-term repaying ability became weak in Shandong (Ke et al. 2014). However, there was almost no evaluation of the hospital terminal quality before and after the reform.

As the reform process of public hospital speeds up, county hospitals achieved further development and certain results in some aspects. Whereas, whether pilot results were in line with the basic goal of reform was a question. In 2013, Guangxi government announced to preliminarily evaluate the reform effectiveness of the first pilot public hospitals. In this paper, the objectives of our study were to evaluate the reform effectiveness of county public hospital from efficiency and quality without measuring all aspects of hospital operations.

## Methods

### Study design and data source

Based on the objectives of county public hospital reform, our research team has tracked and evaluated the effectiveness of health reform from the perspective of efficiency and quality in the following five aspects: (1) the relative operating efficiency of hospitals; (2) the clinical terminal quality; (3) the average medical expense of patients; (4) patient overall satisfaction; (5) medical staff overall satisfaction. Those sample hospitals were General Hospitals, Traditional Chinese Hospitals, and Women and Child Health Care Hospitals from the first pilot seven counties (pilot group) and from another seven non-pilot county public hospitals (non-pilot group), namely Pinxiang, Pingguo, Pubei, Yongfu, Du’an, Hepu and Beiliu county. Non-pilot county public hospitals were selected mainly according to that those hospitals had the same level and similar service quantity and medical circumstance.

A series of questionnaires were designed and the individual interview was adopted to collect insightful viewpoints and assessments of hospital directors, doctors, nurses, patients. Quantitative data from 2010 to 2012 were collected from the questionnaires on 14 sample hospitals, including tangible and intangible assets, health human resources, quantity of health service, bed occupancy rate, the average medical expenses of outpatient and discharged inpatient, and so on. Others were collected via the satisfaction questionnaires of medical staffs, inpatient and outpatient from June 20 to September 30, 2013. Some of our research team took place as a seminar with pilot hospital directors, doctors, nurses to understand their viewpoints towards reform and its effect and evaluation on the reform, each seminar has lasted about 60 min, and their questionnaires of satisfaction were collected at the end of the seminar.

The others collected outpatient’s questionnaires of satisfaction by convenience sampling, while inpatient’s questionnaires by systematic sample according to their hospital computerized medical records. To get high effective response rate, the interpreter was assigned, if necessary, to eliminate language barriers from the participants’ local language or dialect; the respondents were given a full explanation of the research purpose before being invited to participate and then signed the informed consent; each participant was more than sixteen and could independently express their feelings; it usually takes 5 min to finish each anonymous questionnaire.

In total, 14 questionnaires on hospitals were collected with the effective response rate 100%, while 614 and 697 questionnaires on outpatients and inpatients of 14 sample hospitals were collected, with the effective response rate 95.36 and 97.15%, respectively; 185 questionnaires on medical staffs were collected in pilot hospitals with the response rate 95.86%. The questionnaires survey was used to abstract the satisfaction data relevant to the study question. Specifically, the overall satisfaction with health service was determined using the following main questions: for patients, “how did you feel with overall health service?” for medical staffs in pilot hospitals, “how did you feel now about the outcomes of new medical reform? To get clear responses, all of these questions have the following possible responses without setting an “undecided” or “don’t know” option: strongly satisfied, satisfied, dissatisfied, strongly dissatisfied. These were analyzed by assigning each response a score from 4 to 1 (4 = strongly satisfied, 3 = satisfied, 2 = dissatisfied, 1 = strongly dissatisfied).

### Data analysis

The data envelopment analysis (DEA) methodology was used to evaluate the hospital relative operating efficiency with panel data between 2010 and 2012. DEA is not a stochastic and parametric estimation of a hospital’s efficiency wherein a mathematical linear programming problem with multiple inputs and outputs is solved to construct a piece-wise linear best practice frontier (Clement et al. 2008). A detailed description of the DEA method is presented by Seiford and Thrall (1990). The CCR mode, which was proposed by Charnes et al. (1978) and BCC mode which was proposed by Banker et al. (1984), have been widely applied to measure relative efficiency (Hadad et al. 2013; Cheng et al. 2015; Ozcan et al. 1992). According to optimum seeking of literature and previous empirical research (Tlotlego et al. 2010; Kirigia and Asbu 2013; Harrison et al. 2004; Sahin and Ozcan 2000; Akazili et al. 2008; Kawaguchi et al. 2014; Köse et al. 2014; Weng et al. 2009; Kirigia et al. 2004, 2008), three variable modes were used to evaluate the relative operating efficiency of sample hospitals in consideration of the influence of different input and output variables on the evaluating results (Table 1), and the comparison of three modes explained strongly hospital operating efficiency with reducing deviation factor of evaluation.

**Table 1:** Descriptive statistics for DEA model variables between twenty pilot and twenty non-pilot hospitals (${\bar{\text{x}}}$ ± SD)

Variable	Pilot		Non-pilot	
	2010	2012	2010	2012
Inputs				
Actual number of beds	215.45 ± 177.75	224.85 ± 193.38	238.45 ± 220.86	287.00 ± 284.47
Total number of medical staffs	298.30 ± 176.59	350.95 ± 214.06	351.90 ± 354.09	417.70 ± 406.41
Hospital total expenditure	4019.62 ± 3338.76	6151.74 ± 5181.75	5388.50 ± 6306.85	8136.32 ± 9685.01
Fixed assets	4094.23 ± 4956.33	5480.13 ± 6713.60	4470.71 ± 5328.25	5749.02 ± 6390.68
Outputs				
Person-time of outpatient visits	156,206.20 ± 108,077.06	191,422 ± 135,713.20	162,923.50 ± 131,648.16	194,714.00 ± 154,406.64
Number of discharged patients	9619.15 ± 7864.33	12,650.50 ± 10,610.09	10,380.30 ± 10,627.08	14,168.25 ± 14,086.77
Incomes of health service	4294.62 ± 3789.52	6651.62 ± 5758.02	5992.39 ± 7428.47	9226.86 ± 11,033.83

The Ridit analysis was applied to evaluate the clinical terminal quality as an important evaluation index of health service. A non-parametric test, such as Wilcoxon rank sum test, was used to the analysis of satisfaction and t-tests for patient’s medical expenses, *p* value ≤0.05 to indicate statistical significance. All analyses were conducted using SPSS statistical software (version 16.0) and DEAP analytical software (version 2.1).

## Results

### Relative operating efficiency

Table 2: In mode I, input variables was the actual number of beds and the total number of medical staffs, while outputs were person-time of outpatient visits and number of discharged patients. The (mean ± SD) s of the different values of efficiency between 2010 and 2012 were −0.0264 ± 0.0812 (pilot group) and −0.0080 ± 0.1444 (non-pilot group). The data was normally distributed within each group (*p* value_pilot_ = 0.999, *p* value_non-pilot_ = 0.696) and the two population variances were equal at the significant level 0.05 (F = 2.562, *p* value = 0.118). The t-test of two independent-samples resulted in t = −0.495, *p* value = 0.623.

**Table 2:** The comparing result on operational efficiency between pilot and non-pilot hospitals

Modes	Group	${\bar{\text{d}}}_{{2012{-}2010}} \pm {\text{S}}_{\text{d}}$	t	*p* value
I	Pilot	−0.0264 ± 0.0812	−0.495	0.623
	Non-pilot	−0.0080 ± 0.1444		
II	Pilot	0.0424 ± 0.0742	0.855	0.398
	Non-pilot	0.0210 ± 0.0841		
III	Pilot	0.0347 ± 0.0763	0.412	0.683
	Non-pilot	0.0220 ± 0.1141		

In mode II, the input variables were actual number of beds, total number of medical staffs and hospital total expenditure, while outputs were person-time of outpatient visits and number of discharged patients. The (mean ± SD) s of the different values of efficiency between 2010 and 2012 were 0.0424 ± 0.0742 (pilot group) and 0.0210 ± 0.0841 (non-pilot group). The data was normally distributed within each group (*p* value_pilot_ = 0.884, *p* value_non-pilot_ = 0.445) and the two population variances were equal at the significant level 0.05 (F = 0.069, *p* value = 0.795). The t-test of two independent-samples resulted in t = 0.855, *p* value = 0.398.

In mode III, the input variables were the actual number of beds, total number of medical staffs, hospital total expenditure, and fixed assets, while outputs were person-time of outpatient visits, number of discharged patients, and incomes of health service. The (mean ± SD) s of the different values of efficiency between 2010 and 2012 were 0.0347 ± 0.0763 (pilot group) and 0.0220 ± 0.1141 (non-pilot group). The data was normally distributed within each group (*p* value_pilot_ = 0.987, *p* value_non-pilot_ = 0.250) and the two population variances were equal at the significant level 0.05 (F = 2.369, *p* value = 0.132). The t-test of two independent-samples resulted in t = 0.412, *p* value = 0.683.

### Clinical terminal quality

There were four curative effects reflecting the clinical terminal quality of hospitals, namely cured, improved, uncured, and death. According to the number of discharge patients for different clinical terminal quality outcomes in 2010 and 2012 between twenty pilot and twenty non-pilot hospitals, the Ridit value (R) of each group with its 95% CI in different group was shown in Table 3.

**Table 3:** The R value of each group with its 95% CI in different group by the Ridit methodology

Group	R	95% CI
Pilot group		
2010	0.495732	0.495731–0.495733
2012	0.500000	0.499999–0.500001
Non-pilot group		
2010	0.495404	0.495403–0.495405
2012	0.500000	0.499999–0.500001
2010		
Pilot group	0.500000	0.499999–0.500001
Non-pilot group	0.498105	0.498104–0.498106
2012		
Pilot group	0.500000	0.499999–0.500001
Non-pilot group	0.498405	0.498405–0.498406

### Average medical expenses of patient

Table 4: For average medical expenses of outpatients. The difference between 2010 and 2012 was normally distributed within each group (*p* value_pilot_ = 0.528 and *p* value_non-pilot_ = 0.760) and the two population variances were equal at the significant level 0.05 (F = 0.309, *p* = 0.581). The t-test of two independent-samples resulted in t = −1.861, *p* value = 0.077. The difference between the two means was −9.3165 yuan (95% CI = −19.7005 yuan to 1.0675 yuan).

**Table 4:** The comparing result on the effect of controlling medical expense of outpatient and inpatient

Groups		${\bar{\text{d}}}_{{2012{-}2010}} \pm {\text{S}}_{\text{d}}$	t	*p* value
Outpatient	Pilot group	13.0895 ± 17.1321	−1.816	0.077
	Non-pilot group	22.4056 ± 15.2549		
Inpatient	Pilot group	527.2730 ± 243.6208	−1.654	0.106
	Non-pilot group	715.8475 ± 447.9602		

For average medical expenses of inpatients. The difference between 2010 and 2012 was normally distributed within each group (*p* value_pilot_ = 0.709 and *p* value_non-pilot_ = 0.461) and the two population variances were equal at the significant level 0.05 (F = 1.535, *p* value = 0.223). The t-test of two independent-samples resulted in t = −1.654, *p* value = 0.106 (Table 4). The difference between the two means was −188.5745 yuan (95% CI = −419.3996 yuan to 42.2506 yuan).

### Patient overall satisfaction

Sociodemographic characteristics of the investigated patients in pilot and non-pilot county hospitals are shown in Table 5.

**Table 5:** Sociodemographic characteristics of the investigated patients in pilot and non-pilot county hospitals

Characteristic	Pilot group	Non-pilot group	Total
	N (%)	N (%)	N (%)
Gender			
Male	277 (39.91)	266 (43.11)	543 (41.42)
Female	417 (60.09)	351 (56.89)	768 (58.58)
Age			
16–25	89 (12.82)	106 (17.18)	195 (14.87)
26–35	121 (17.44)	113 (18.31)	234 (17.85)
36–45	143 (20.61)	125 (20.26)	268 (20.44)
46–55	195 (28.10)	162 (26.26)	357 (27.23)
56+	146 (21.04)	111 (17.99)	257 (19.60)
Ethnic group			
Han	218 (31.41)	231 (37.44)	449 (34.25)
Zhuang	244 (35.16)	217 (35.17)	461(35.16)
Yao	83 (11.96)	96 (15.56)	179 (13.65)
Miao	97 (13.98)	58 (9.40)	155 (11.82)
Dong	36 (5.19)	13 (2.11)	49 (3.74)
Others	16 (2.31)	2 (0.32)	18 (1.37)
Educational level			
Primary school and below	170 (24.50)	165 (26.74)	335 (25.55)
Secondary school	263 (37.90)	251 (40.68)	514 (39.21)
Senior secondary school	157 (22.62)	138 (22.37)	295 (22.50)
Junior college	92 (13.26)	53 (8.59)	145 (11.06)
Bachelor’s degree or above	12 (1.73)	10 (1.62)	22 (1.68)
Health insurance			
New rural cooperative medical system	413 (59.51)	367 (59.48)	780 (59.50)
Urban residents basic medical insurance	158 (22.77)	164 (26.58)	322 (24.56)
Urban employee basic medical insurance	43 (6.20)	65 (10.53)	108 (8.24)
No medical insurance	39 (5.62)	16 (2.59)	55 (4.20)
Others	41 (5.91)	5 (0.81)	46 (3.51)
Treatment type			
Outpatient	326 (46.97)	288 (46.68)	614 (46.83)
Inpatient	368 (53.03)	329 (53.32)	697 (53.17)

Table 6: The analysis sample consisted of a total of 614 outpatients. Of these, 30 (9.30%) were strongly satisfied, 268 (82.21%) were satisfied, 23 (6.98%) were dissatisfied, and 5 (1.53%) were strongly dissatisfied in pilot hospitals, while 45 (15.79%) were strongly satisfied, 198 (68.75%) were satisfied, 38 (13.16%) were dissatisfied, and 7 (2.43%) were strongly dissatisfied in non-pilot hospitals. The rank sum test for the comparison between two independent samples of an ordinal variable resulted in Z = −0.139, *p* value = 0.889.

**Table 6:** The comparing results of outpatient and inpatient overall satisfaction between pilot and non-pilot groups in 2013

Group	Z	*p* value
Outpatient		
Pilot group	−0.139	0.889
Non-pilot group		
Inpatient		
Pilot group	−4.671	0.000
Non-pilot group		

Another analysis sample consisted of a total of 697 inpatients. Of these, 85 (23.10%) were strongly satisfied, 254 (69.02%) were satisfied, 28 (7.70%) were dissatisfied, and 1 (0.27%) were strongly dissatisfied in pilot hospitals, while 34 (10.33%) were strongly satisfied, 251 (76.29%) were satisfied, 40 (12.06%) were dissatisfied, and 4 (1.22%) were strongly dissatisfied in non-pilot hospitals. The rank sum test for the comparison between two independent samples of an ordinal variable resulted in Z = −4.671, *p* value = 0.000.

### Medical staff overall satisfaction

The analysis sample consisted of a total of 185 medical staffs. Of these, 30 (16.22%) were strongly satisfied with the outcomes of new medical reform, while 28 (15.32%) satisfied, 82 (44.14%) dissatisfied, and 45 (24.32%) strongly dissatisfied in pilot hospitals. It is shown that nearly 68.46% medical staffs were not dissatisfied with the outcomes in 2013.

## Discussion

As can be seen from the above analysis, the difference in each group of each mode was not statistically significant (*p* value > 0.05), we could not draw the conclusion that there was a difference on the relative operating efficiency of sample hospitals between pilot and non-pilot groups. The relative operational efficiency of the first pilot county public hospitals in 2012 was more than that in 2010 except for mode I. The findings showed that the reform could not have improved fully county public hospital operational efficiency. According to service populations, health investment of most county hospitals was relative redundant, and the expansion of hospital scale was too large, which results from the increasing health investments of all levels of government since 2011.

The clinical terminal quality of hospitals in 2012 was better than that in 2010 either in pilot or non-pilot group (*p* value < 0.05). Meanwhile, the pilot hospitals’ quality was better than that in non-pilot groups either in 2010 or 2012 (*p* value < 0.05). The findings showed that the clinical terminal quality of both pilot and non-pilot group all got improvement with the improvement of medical technology and service ability. However, county public hospital reform did not significantly play a due good role in improving the medical services quality in pilot group which is one of the reform’s goals. One of the important reasons was China health reform policy was paying more attention to inputs rather than outputs for hospital and lack of evaluation and supervision on hospital running or operation. Besides, the decision-making department of public health made the decision of how much should be in each hospital by experience management without attaching importance to equity and efficiency, as well as quality.

From analysis results of average medical expenses of patient, the county public hospital reform has taken an effect on slowing the growth of medical expense. However, there was not a difference on the effect of controlling medical expense of outpatient between pilot and non-pilot groups (*p* value > 0.05), while the difference was significant in inpatient (*p* value < 0.05). This is mainly because medical insurance policy improved the reimbursement ratio of inpatients and the usage efficiency of medical insurance funds, pilot hospitals carried out zero rates of sales drug (ZRSD) varieties scope and classified diagnosis and treatment (CDT) as well as medical payment reform (MPR).

For satisfaction, patients were satisfied with overall health service in 2013, but the difference between pilot and non-pilot outpatients was not statistically significant (*p* value > 0.05), while the pilot group’ satisfaction was better than non-pilot group’s (*p* value < 0.05) in that inpatients stayed in hospital for a short period of time with preferably experiencing medical services and environment improved by the reform. Nearly 68.46% medical staffs were dissatisfied with the outcomes of the reform mainly because their working pressures kept high while incomes were not to be improved from in-depth interview.

### Limitations

For this study, there are several limitations needed to be mentioned. For one thing, those sample hospitals located in remote areas of China, the local dialect led us to collected questionnaires of some patients with the help of a nurse as an interpreter and maybe reduce the reliability of the questionnaire.

For another, our evaluation just for about 1 year after the reform of the first pilot country public hospitals in Guangxi led to reducing the stability and objectivity of the evaluating results, the set of non-pilot group could not fully conform to the requirement of the intervention experiment, which might lead to some bias. The findings only reflected the reform effectiveness of Guangxi except for other areas of China.

In further research design, the sample can be extended to include all pilot country public hospitals in Guangxi or in another region, with respective efficiency and quality analysis for comparison. Meanwhile, alternative methods are encouraged to be applied for evaluating the reform effect.

## Conclusion

The county public hospital reform does not completely achieve policy objectives in Guangxi. Its investment improved the developing conditions of public hospital, but hospital operating efficiency, clinical terminal quality and outpatient’s satisfaction were not to be consequently improved, and medical staffs were dissatisfied with the outcomes of the reform. The reform has slowed the growth rate of average medical expenses of patient, but the total medical expenditure is still growing, that is to say, the patient’s burden of disease is still very heavy. In short, the county public hospital reform has not completely succeeded in Guangxi. Therefore, starting the second pilot county public hospital reform and even in an all-round way would be unwise and hasty before dealing well with some questions of the first pilot county public hospital reform. Some supporting measures should be taken to further improve health investments mode, medical technology level, medical service quality, and the performance pay system of medical staff. Besides, it provided an insight into the performance of Chinese county hospitals during the reform process, which can assist policymakers in choosing the best regulatory framework for the ongoing hospital reform process.
